# Determinants of the number of antenatal visits in a metropolitan region

**DOI:** 10.1186/1471-2458-10-527

**Published:** 2010-09-01

**Authors:** Katrien Beeckman, Fred Louckx, Koen Putman

**Affiliations:** 1 Department of Medical Sociology and Health Sciences, Vrije Universiteit Brussel, Faculty of Medicine and Pharmacy, Laarbeeklaan 103, 1090 Brussels, Belgium; 2 Interuniversity Centre for Health Economics Research, Vrije Universiteit Brussel, Faculty of Medicine and Pharmacy, Laarbeeklaan 103, 1090 Brussels, Belgium

## Abstract

**Background:**

Antenatal care has a positive effect on pregnancy, both clinically and psychologically, but consensus about the optimal number of antenatal visits is lacking. This study aims to provide insight into the dynamics of the number of antenatal visits a woman receives. Independent effects of predisposing, enabling and pregnancy-related determinants are examined.

**Methods:**

Women were recruited in nine clinical centres in the Brussels Metropolitan region. Antenatal care use was measured prospectively. A Poisson regression model was applied to measure the independent effect of individual determinants on the number of antenatal visits.

**Results:**

Data on antenatal care trajectories in 333 women were collected. The multivariate analyses showed that women with a Maghreb or Turkish origin had 14% fewer visits compared with European (EU15) women. More highly educated women had 22% more visits compared with those with a low education. Women with a high income had 14% more antenatal visits compared with those with a low income. Fewer antenatal visits were observed in multiparae (15%), women initiating care after 14 weeks of gestation (31%), women without medical risks during the pregnancy (12%) and in women with a continuity of care index of 50% or more (12%). More visits were observed in delivering after week 37 (22% increase).

**Conclusions:**

Predisposing and enabling factors have to be considered when antenatal care programmes are evaluated in a metropolitan area. Variations in the number of antenatal visits show that socially vulnerable women are more at risk of having fewer visits.

## Background

Antenatal care is generally considered to have a positive effect on the health of both mother and baby [1-4]. As well as medical follow-up, advice and information is given and treatment can be provided when needed [1]. Antenatal care not only affects the pregnancy clinically, it also has a psychological effect, preparing women for childbirth and motherhood [5,6]. Guidelines in antenatal care are diverse and not entirely evidence-based. For example, the advised number of antenatal visits differs considerably between Western countries [7-14] and ranges from a minimum of six in the Netherlands [8] to fifteen in Finland [14]. In Belgium, ten visits for primiparae and about seven for multiparae are advised [13]. Although there is no consensus about the optimal number of antenatal visits, it is proved that inadequate antenatal care is related to a worse pregnancy outcome [14-16]. It is important for health policy makers to understand what factors have an influence on a reduced number of antenatal visits. Analysis of utilization of healthcare services is frequently based on the behavioural model developed by Andersen and Newman [17]. This model has been widely used in analyses of other health services disciplines (eg breast cancer screening [18,19] or studies in care for the elderly [20,21]) but only a few studies have applied it when examining the number of antenatal visits [22,23].

**Figure 1:**
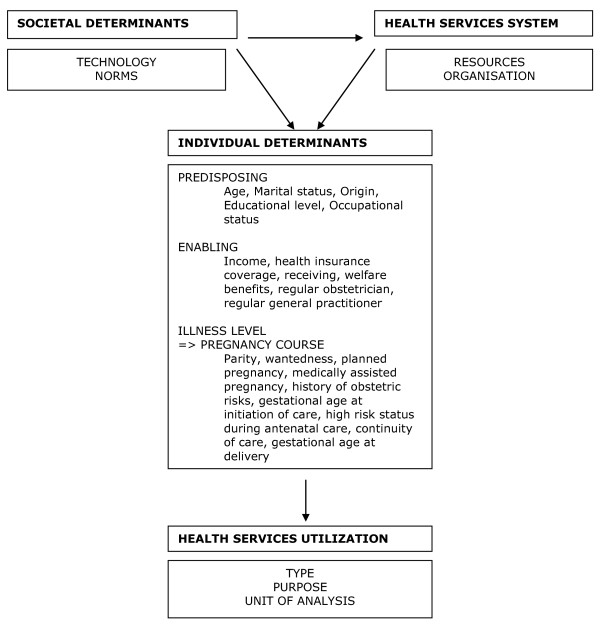
**outline of the behaviour model by Andersen and Newman **[17], **reflecting individual determinants included in the analyses**.

This conceptual framework helps a better understanding of the relationship between societal determinants, determinants of the health care system and individual determinants on the use of health care [17]. Figure 1 represents the framework as defined by Andersen and Newman [17,24]. With regard to the individual determinants, the predisposing component refers to the characteristics of the person and includes demographic, social position and attitudinal variables [17,24]. The enabling component consists of conditions that make health care use available to the person [17,24]. The third component, 'illness level', is most directly related to health care use and comprises perceived illness and diagnosed illness [17,24]. In the context of our study, 'illness level' can be translated to pregnancy history and the current course of the pregnancy.

Our study examines the use of antenatal care in the Brussels metropolitan region. It can be assumed that the health care context was the same for all women in this region and that the organization of antenatal care was also similar. Consequently, the effect of variation in health care systems as defined by Andersen and Newman is limited. We therefore decided to focus on individual determinants which influence health care use directly [17,24]. The aim of this study is to measure the effect of predisposing, enabling and pregnancy-related factors on the use of antenatal care.

## Methods

### Data collection

A prospective observational study of antenatal care use was conducted in the Brussels Metropolitan region. Women were recruited consecutively in nine of the eleven antenatal clinical centres spread over the region. During pregnancy each woman is referred to one of these centres for their ultrasound screening. The inclusion criteria were: having a gestational age of less than sixteen weeks or attending the third antenatal visit or less, residing in the Brussels Metropolitan Region, aged over eighteen years and being able to speak Dutch, French, English, Turkish or Arabic (these languages cover 95.5% of the Brussels population [25]). The exclusion criteria were: multiple pregnancies, existing medical problems (heart disease, diabetes, hypertension or renal diseases for instance), no informed consent and not being reachable by phone. We obtained ethical approval from all participating sites and from the Ethics Committee of the University Hospital UZ Brussel, prior to the start of the study.

At the moment of recruitment, a first questionnaire about personal characteristics and pregnancy history was filled out. Any antenatal visit prior to recruitment was documented by structured interview. Additionally, all participating women received a diary, developed to record every antenatal visit in a standardised manner. The researcher explained how to use the diary and a manual was provided. Various alternatives of data collection were considered, like use of medical files or data registers. These options were lacking the level of detail required or were not reliable for our study. Use of a diary enabled us to record every visit, even when the women change from care provider or care setting. Bimonthly telephone follow-up interviews were conducted to record the antenatal care use and to verify the completeness of the data.

### Variables

An antenatal visit was defined as a contact with a health care professional to follow up pregnancy. Antenatal care can be provided by an obstetrician, midwife or generalist. If the woman visited more than one professional at the same place on the same day, this was counted as one visit. Separate visits to the laboratory for a blood sample and/or urine test were not counted as an antenatal visit.

The total number of visits was analysed in association with predisposing, enabling and pregnancy related characteristics and presented in figure 1. The mother's age, marital status, origin, educational level and activity status were the predisposing variables examined.

The following enabling factors were considered in the analyses: having health insurance coverage, receiving welfare benefits, having a regular obstetrician and/or regular generalist, equivalent income. Equivalent income was calculated using the modified Organization for Economic Co-operation and Development (OECD) scale [26], based on the monthly household income and household composition. The women were regrouped into three categories. The lowest income group was defined at 60% or less than the median equivalent national income for Belgium [27]. This threshold of 60% is the at-risk-of-poverty threshold [28]. Equivalent incomes between 60% and 120% of that threshold were considered as moderate incomes. An income above 120% of the median national equivalent income was defined as high income. Data on the net monthly income of thirty one women (9.3%) were missing. This was more often the case in women active in the labour market and those more highly educated (Chi^2 ^analyses; available upon request). Within the group of more highly educated, active women, missings occurred at random. Multiple imputation methodology was applied to estimate missing data. Imputation was performed multiple times to create 5 complete data sets. In conducting our analyses, we used the Poisson regression techniques in the 5 imputed data sets. We then combined the results via the MIANALYZE procedure in SAS version 9.1 (SAS Institute, Cary, NC) to obtain the overall parameter estimates [29,30]. Educational level, activity status and health insurance coverage were the predictors used for the imputation.

A third group of variables referred to pregnancy-related determinants. Parity, the pregnancy being planned, the pregnancy being desired, medically assisted pregnancy, history of obstetric risk (previous preterm birth, low birth weight, miscarriage, still birth, admission to neonatal care unit), high risk status during antenatal care (hypertension, pre-eclampsia, gestational diabetes mellitus, preterm contractions, hospital admission or anaemia), gestational age at initiation of care, and continuity of carer, were included in the analysis. Continuity of care was measured by the COC index [31,32]. This index counts the number of visits to each different health care provider compared to the total number of visits.

### Statistical analysis

First, the number of antenatal visits was analysed bivariatly using Mann-Withney or Kruskall-Wallis tests. Second, a multivariate Poisson regression model was used to examine the determinants for differences in the number of antenatal visits. A Poisson model is standard when analyzing count data, especially when skewed distributions are observed [33,34], such as the number of antenatal visits. This multivariate model is used to quantify the independent effects of predisposing, enabling and pregnancy-related factors after adjusting for confounding factors. As this was an exploratory study, we aimed to include all variables and choose for a backwards model as the best option. Corresponding to the health behavior model of Andersen and Newman [17,24], all predisposing variables were considered first. Variables that were not significant at p < 0.05 were subsequently omitted from the model, starting with the variable with the highest p-value. This cycle was repeated until all variables were significant at the p < 0.05 level. In the next round, the enabling factors were considered, with the selected predisposing variables fixed in the model. Again the least significant variable was left out and the model rerun to select a set of significant variables. In the third round, the pregnancy-related factors were studied, controlling for the variables in the first two rounds. Unadjusted and adjusted differentials in antenatal visits and 95% confidence intervals (CIs) were calculated for all women in the study. The distribution of residuals and their relationship with the linear predictor, as well as the deviance reduction, were all examined in order to test goodness-of-fit of the model. We used SAS 9.1 for all the analyses.

## Results

### Characteristics of the study sample

**Figure 2:**
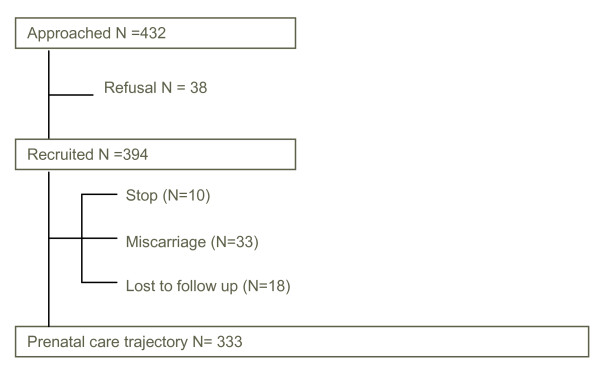
**recruitment scheme**.

Four hundred and thirty-two women corresponded to the inclusion criteria (figure 2); there were thirty eight refusals. The main reasons not to participate in the study were lack of interest and disapproval by the partner. The women who refused did not differ from the women in the study regarding age and origin (Fisher Exact, p = 0.60 & p = 0.43 respectively). In total, three hundred ninety-four women were included in the study and sixty one dropped out. Ten women stopped their participation after the first telephone call, thirty three because of miscarriage; eighteen were lost to follow-up, despite additional verifications with the clinical centres and reminders being sent. Compared with the final sample, significantly more Maghreb, Turkish and women of another origins dropped out (p = 0.007). The proportion of women active in the labor market was significantly lower in the drop-out group compared with the study sample (p = 0.035). (Results not shown but available on request).

Data on the complete antenatal care trajectory of three hundred thirty-three women were collected. Table 1 represents the predisposing, enabling and pregnancy-related characteristics in our sample. Concerning age, 79.5% of the women were aged between twenty one and thirty five. Of the women, 40.3% had the Belgian or other European (EU15) origin and 33.6% had a Maghreb or Turkish origin. 14.7% did not finish secondary school. 54.7% of the women were inactive on the labor market. For the enabling characteristics, we observed that 27.3% of the women had a low equivalent income. Regarding health insurance, 93.4% of the women had health insurance coverage and 9.3% received welfare benefits. The majority (52%) had no regular obstetrician.

**Table 1:** characteristics of the study sample (N = 333) and differences in the number of antenatal visits between subgroups

		Study Sample		Total number of Antenatal visits		
		**N**	**%**	**Median**	**P25-P75**	**P**

**Predisposing characteristics**						

Age* ^a^	<= 20	14	4.2	11	11-13	NS
	21-35	265	79.5	12	10-15	
	>35	53	15.9	13	10-15	
Marital Status ^b^	Co-habiting	302	90.7	11	9-14	NS
	Single	31	9.3	12	10-15	
Origin^a^	15 oldest EU countries^c^	134	40.3	13	10-17	< 0.001
	Maghreb and Turkey	112	33.6	11	9-13	
	Sub-Saharan African	49	14.7	13	9-14	
	Other	38	11.4	12	10-15	
Educational level ^a^	Not finished secondary school	49	14.7	10	9-12	< 0.001
	Secondary school	150	45.0	11	9-13	
	Higher education	134	40.2	14	11-18	
Occupational status ^b^	Active on labour market	151	45.3	13	10-17	< 0.001
	Not active on the labour market	182	54.7	11	9-14	

**Enabling characteristics**						

Equivalent income^a,d^	Low	91	27.3	11	9-13	< 0.001
	Moderate	164	49.2	11.5	10-14	
	High	78	23.4	15	12-18	
Health Insurance Coverage ^b^	Yes	311	93.4	11	10-14	NS
	No	22	6.6	12	10-15	
Receiving welfare benefits* ^b^	Yes	31	9.3	12	10-15	NS
	No	301	90.4	11	9-13	
Regular obstetrician ^b^	Yes	160	48.0	12	10-14	NS
	No	173	52.0	12.5	10-16	
Regular General Practitioner ^b^	Yes	206	61.9	11	9-14	0.042
	No	127	38.1	13	10-16	

**Pregnancy related characteristics**						

Parity ^b^	Primiparae	128	38.4	13.5	11-17	< 0.001
	Multiparae	205	61.5	11	9-14	
Wanted pregnancy* ^b^	Later or not	52	15.6	11.5	9.5-13	NS
	Earlier or right time	280	84.1	12	10-16	
Planned pregnancy ^b^	Yes	257	77.2	11	9-13	0.002
	No	76	22.8	12	10-16	
Medical assisted pregnancy* ^b^	Yes	15	4.5	12	10-15	NS
	No	317	95.2	12	10-16	
History of obstetric risks ^b,e^	Yes	92	27.6	12	10-16	NS
	No	241	72.4	12	10-14	
Gestational age at initiation of care ^b^	Before week 14	316	99.5	12	10-15	< 0.001
	After week 14	17	0.05	9	6-10	
High risk status during antenatal care^b,f^	Yes	51	15.3	12	10-15	0.023
	No	282	84.7	14	10-17	
Continuity of Carer Index ^b,g^	<50%	253	76.0	12	10-16	0.002
	>= 50%	80	24.0	11	9-13	
Gestational age at delivery ^b^	<37 weeks	24	7.2	10	9-13	0.016
	>= 37 weeks	309	92.8	12	10-15	

*1 missing **2 missing

NS Not Significant

P25-P75 Percentile 25-Percentile 75

^a ^Kruskal-Wallis test

^b ^Mann-Whitney test

^c ^Belgium, Denmark, Finland, France, Germany, Greece, Ireland, Italy, Luxembourg, the Netherlands, Austria, Portugal, Spain, Sweden and the United Kingdom

^d^Σ incomes in the household/(1+(a*0.5)+(c*0.3)) with a: number of adults living in the same household of the wife (wife not included), c: number of children under the age of 18 living in the same household (OECD scale)[26]

^e ^history of preterm baby, low birth weight, miscarriage, still birth or admission on Neonatal Intensive Care Unit

^f ^hypertension, preeclampsia, gestational diabetes mellitus, preterm contractions, hospitalization, anaemia

^g ^Bice index or Continuity of Care index: COC = $\frac{\sum n_{j}^{2}-n}{n\left(n-1\right)}$ (Bice, 1977) [31,32]

n_j _is the total number of visits to a provider

n is the total number of visits

For the pregnancy related characteristics, 38.4% of the women were primiparae. The pregnancy was not planned in 22.8% of cases. 27.6% of the women had a history of obstetric risk, with 17.1% having a previous miscarriage and 0.9% having a previous stillbirth. Other obstetric risks in previous deliveries were the experience of a previous preterm birth (15.3%), a previous delivery of a low birth weight baby (6.3%) or an admission to an intensive neonatal care unit (7.8%) (results not shown in the table). High risk status during the antenatal care period (15.3%) was most often related to hospital admission (7.2%). Other reasons were preterm contractions (3.6%), followed by hypertension (3%), gestational diabetes mellitus (3%) and pre-eclampsia (2.1%) (results not shown). 24% of the women saw the same health care provider in more than half of their antenatal visits (COC >= 50%). Concerning pregnancy outcome, we observed 7.2% that delivered before 37 weeks of gestation.

Overall, the median number of antenatal visits was 12 (Percentile 25-Percentile 75: 10-15) with a range between three and forty one (results not shown).

Bivariate analyses showed significant differences in number of visits for origin (p < 0.001), education (p < 0.001) and occupational status (p < 0.001) (table 1). Concerning the enabling determinants we observed a relationship with equivalent income (p < 0.001). Furthermore, women without a regular generalist had fewer antenatal visits than did those who had one (p = 0.042).

When examining the pregnancy-related determinants, we found a significantly lower number of antenatal visits in multiparae (p < 0.001). Women with an unplanned pregnancy also had significantly fewer visits (p = 0.002). Further, women who initiated care after week fourteen made significantly fewer antenatal visits (p < 0.001). No medical risk status during antenatal care (p = 0.023) and a continuity of carer index of 50% or above (p = 0.002) was also associated with a significantly lower number of antenatal visits. Additionally, more antenatal visits were observed in women with a full term baby (>= 37 weeks) (p = 0.016).

### Determinants of the number of antenatal visits

The Poisson regression model (table 2) shows the significant predisposing, enabling and pregnancy-related factors of the number of antenatal visits. The following characteristics were independently associated with the number of antenatal visits received: origin, educational level, equivalent income, parity, gestational age at initiation of care, high risk status during antenatal care, gestational age at delivery and continuity of care index. Women of Maghreb or Turkish origin had 14% fewer antenatal visits compared with those from Europe (EU15) (adjusted visit ratio 0.86, 95% CI 0.79-0.92). More highly educated women had 22% more antenatal visits compared with those with lower levels of education (adjusted visit ratio 1.22, 95% CI 1.10-1.35). Furthermore one enabling factor remained when controlling for confounders. Women with a high equivalent income had 14% more antenatal visits, and those with a moderate income had 9% more visits compared with those with a low income (adjusted visit ratio 1.14, 95% CI 1.02-1.27 and 1.09, 95% CI 1.01-1.19, respectively). When controlling for predisposing and enabling factors, the following-pregnancy related factors were observed: multiparae had 15% fewer visits than primiparae (adjusted visit ratio 0.85, 95% CI 0.80-0.91). Women who initiated care after week fourteen had 31% fewer antenatal visits (adjusted visit ratio 0.69, 95% CI 0.58-0.82). Absence of high risk status during pregnancy was associated with 12% less antenatal visits (adjusted visit ratio 0.88, 95% CI 0.81-0.96). A delivery after 37 weeks of gestation lead to 22% more antenatal visits compared to a preterm delivery (adjusted visit ratio 1.22, 95% CI 1.07-1.39). After controlling for all independent variables, women with a COC index of more than 50% had 12% less antenatal visits compared with women with less continuity of care in their antenatal care trajectory (adjusted visit ratio 0.88, 95% CI 0.82-0.94).

**Table 2:** Poisson regression describing differences in adjusted number of antenatal visits

Variable		Adjusted visits	**Adjusted visit ratio**^**f**^	**95%CI**^**g**^	p-value
**Predisposing characteristics**					

Origin	EU 15^a^	10.60 ^e^	1 ^e^		
	Maghreb and Turkey		0.86	0.79-0.92	0.0001
	Sub-Saharan African		0.94	0.85-1.03	0.2059
	Other		0.94	0.85-1.05	0.3193
Educational level	Not finished secondary school	9.18 ^e^	1 ^e^		
	Secondary school		1.03	0.93-1.14	0.5198
	Higher education		1.22	1.10-1.35	0.0001

**Enabling characteristics**					

Equivalent income ^b^	Low	9.20^e^	1 ^e^		
	Moderate		1.09	1.01-1.19	0.0353
	High		1.14	1.02-1.27	0.0196

**Pregnancy related characteristics**					

Parity	Primiparae	10.72 ^e^	1 ^e^		
	Multiparae		0.85	0.80-0.91	<0.0001
Gestational age at initiation of care	Before week 14	11.89 ^e^	1 ^e^		
	After week 14		0.69	0.58-0.82	<0.0001
High risk status during antenatal care^c^	Yes	10.56 ^e^	1 ^e^		
	No		0.88	0.81-0.96	0.0036
Gestational age at delivery	Before week 37	8.98 ^e^	1 ^e^		
	After week 37		1.22	1.07-1.39	0.0028
Continuity Of Care index^d^	COC <50%	10.56 ^e^	1 ^e^		
	COC >= 50%		0.88	0.82-0.94	0.0009

^a ^Belgium, Denmark, Finland, France, Germany, Greece, Ireland, Italy, Luxembourg, the Netherlands, Austria, Portugal, Spain, Sweden and the United Kingdom;

^b^Σ incomes in the household/(1+(a*0.5)+(c*0.3)) with a: number of adults living in the same household of the wife(wife not included), c: number of children under the age of 18 living in the same household (OECD scale)[26];

^c ^hypertension, pre-eclampsia, gestational diabetes mellitus, preterm contractions, hospitalization, anaemia

^d ^Bice index or Continuity of Care index: COC = $\frac{\sum n_{j}^{2}-n}{n\left(n-1\right)}$ (Bice, 1977)[31,32]

n_j _is the total number of visits to a provider

n is the total number of visits;

^e ^baseline;

^f ^Adjusted for effects of origin, educational level, equivalent income, parity, gestational age at booking, risk status during antenatal care, gestational age at delivery and Continuity of carer index;

^g ^CI = Confidence interval.

## Discussion

In this study, predisposing, enabling and pregnancy-related variables were considered in measuring the number of antenatal visits in a metropolitan area. Besides origin, educational level and equivalent income, parity, gestational age at initiation of care and at delivery, high risk during antenatal care and continuity of care index were related with the number of antenatal visits.

The influence of predisposing determinants on the number of antenatal visits show a trend towards fewer antenatal visits in socio-economically disadvantaged women. Petrou et al. [35] also observed the importance of origin in relation to the number of antenatal visits. White British women had the highest number while Pakistani women had the lowest number. The study of Hildingsson et al. [36] found no relationship with origin. In contrast with our findings, they found that more highly educated women belonged more often to the group receiving fewer antenatal visits compared with the standard schedule. LaVeist et al. [22] found a relationship between educational level and the number of antenatal visits which was stronger in African American women. Age was not a predisposing determinant for the number of antenatal visits in our study, which was similar to the findings of LaVeist et al. [22] and Trinh et al. [23]. Other studies show different results in the number of visits with increasing age [35,36]. Further Blondel and Marshall [37] found that women who received a maximum of four antenatal visits were more often single. Our study, together with studies in the US [22], Asia [23] and Sweden [36] found no association between marital status and the number of visits.

Predisposing determinants have a substantial independent influence on the number of antenatal visits and highlight groups at risk of receiving fewer antenatal visits. Unfortunately predisposing factors are hard to modify [17]. To change these determinants would require change at a societal level and the optimization of antenatal care programmes would also be needed. Special attention needs to be paid to women with lower levels of education and to certain cultural groups.

Income was the single independent enabling factor associated with the number of antenatal visits. The Vietnamese study [23] was the only one that also included the effect of income as an enabling factor. It found that the highest income group had significantly more chance of having at least three visits compared with the lowest income group. Trinh et al. [23] found more enabling determinants of the number of antenatal visits, and identified the independent effect of having health insurance. Women who were insured were more likely to have three antenatal visits or more. This incongruence with our study may be due to differences in the effects of having health insurance in Asia compared with Europe. Blondel and Marshall [37], in France found that women with fewer than four antenatal visits were more likely to be uninsured. In this study [37] the number of women without health insurance coverage was higher compared with our findings, ranging from 13.6% in French women aged 20 or older to 71.3% in foreign women; this suggests that health insurance coverage can be a proxy for income in their study and might be the underlying cause of the difference with our findings.

In an optimal model explaining health care use, the enabling factors should have minimum influence on the distribution in health services use [17], as this would secure equal availability of health care services. A low equivalent income however was associated with a lower number of antenatal visits.

With regard to pregnancy-related determinants, the differences in the number of antenatal visits related to parity were in line with antenatal care guidelines. More visits are advised in primiparae [13]. Further, women who started care after week fourteen had 32% fewer antenatal visits; this seems logical as some visits are scheduled in the first trimester. Another difference in the number of visits was observed relating to medical risks. Women with medical risks during the course of the pregnancy made 12% more visits compared with those without medical risks during pregnancy. This indicates that the number of visits is influenced by the increased needs that arise during pregnancy. Our study however does not allow us to examine whether the augmentation of the quantity of care was adequate and resulted in better outcomes. The relationship between the number of antenatal visits and gestational age at delivery was as expected, the guideline [13] suggests one or two additional visits after week 37.

A higher number of antenatal visits in primiparae and women with medical risks during pregnancy were also found in the studies of Hildingsson et al. [36] and Petrou et al. [35]. The association between initiation of antenatal care and the number of visits was confirmed by studies in the UK [35] and Asia [23]. A history of medical risks did not seem to be related to the number of antenatal visits in Brussels or Sweden [36]. However, the study in the UK found that women with a high risk status at booking had slightly more visits [35]. We should clarify that the definition of 'history of medical risks at booking' did not cover exactly the same elements in all three studies. The study of Hildingsson [36] only considered two elements: previous miscarriage and previous stillbirth. Women with medical disorders (eg cardiac disease, renal diseases) were excluded from our study, but were considered as high risk status at booking in the study of Petrou et al. [35].

Our study demonstrated that continuity of health care provider was associated with a reduced number of antenatal visits. The study of Petrou et al. [35] reported the opposite, with increased fragmentation leading to fewer antenatal visits. This remarkable difference might be due to the use of a different index. The COC index corrects for the number of visits to each different provider, while the index used by Petrou et al. [35] only divides the number of carers seen by the total number of visits. The COC index will be lower when for example a woman had fifteen visits to three different providers each for five times, compared with one who visited three providers ten, two and three times respectively. This distinction is not made in the index used in the UK study [35].

Some limitations in our study need to be addressed. First, our findings are based on self-reported data. Although events during pregnancy are considered as major life events and are therefore likely to be remembered [38] the number of visits recalled might differ from the actual number. To minimize this discrepancy, recall bias was reduced by performing bimonthly follow-up calls. Second, the sample of our study is rather small. A balance needed to be found between the level of detail of the data collection and the number of cases to be included. However, additional analyses showed that the mean number of antenatal visits in our sample was comparable with the number of consultations in the whole of the Brussels Metropolitan Region (12.9 and 13.3, respectively) [39]. These numbers are comparable to those in other European countries [14,35,36]. To our knowledge, this is the first study in Europe in which individual factors at all three levels of the behaviour model were considered when examining the number of antenatal visits. Also the study is original in its prospective design; provision of bimonthly follow-up ensures accuracy of gathered data.

## Conclusions

Until now, international consensus about the number of antenatal visits has been lacking and the definition of optimal antenatal care differs between guidelines [40]. Therefore, our study focused more on the identification of women at risk of having fewer antenatal visits rather than on the exact number of visits. This approach offered insights into the dynamics behind variations in the number of antenatal visits, based on the behavioural model. Analysis of predisposing, enabling and pregnancy-related determinants of the number of antenatal visits received showed barriers to service use. Our study suggests that variations might be reduced when health care providers pay special attention to socially vulnerable women. Attention should be given to non-European (EU15) women, those with low educational levels and those with low income. These women are at risk of fewer antenatal visits, independently of other factors. This information is valuable for policy makers who develop and evaluate antenatal care programmes. Future research on determinants of other elements of antenatal care, such as initiation of care and content of care, can ascertain whether these dynamics in variations in the amount of antenatal care received remain the same.

## Competing interests

The authors declare that they have no competing interests.

## Authors' contributions

KB contributed to the conception and design of the study, gathered the data, contributed to the analysis and interpretation of data and wrote the article. FL contributed to the conception and design of the study, interpreted the data and revised the article critically for intellectual content. KP contributed to the conception and design of the study, participated in the analysis and interpreting of data, and participated in the drafting and revising of the article. All authors read and approved the final manuscript.

## Pre-publication history

The pre-publication history for this paper can be accessed here:

http://www.biomedcentral.com/1471-2458/10/527/prepub
